# Video-Laparoscopic versus Open Surgery in Obese Patients with Colorectal Cancer: A Propensity Score Matching Study

**DOI:** 10.3390/cancers13081844

**Published:** 2021-04-13

**Authors:** Cinzia Bizzoca, Roberta Zupo, Fabrizio Aquilino, Fabio Castellana, Felicia Fiore, Rodolfo Sardone, Leonardo Vincenti

**Affiliations:** 1 General Surgery Unit “Ospedaliera”, University Hospital “Policlinico” of Bari, 70124 Bari, Italy; f.fiore27@studenti.uniba.it (F.F.); leonardo.vincenti@policlinico.ba.it (L.V.); 2 Unit of Research Methodology and Data Sciences for Population Health, National Institute of Gastroenterology “Saverio de Bellis”, Research Hospital, Castellana Grotte, 70013 Bari, Italy; roberta.zupo@irccsdebellis.it (R.Z.); fabio.castellana@irccsdebellis.it (F.C.); rodolfo.sardone@irccsdebellis.it (R.S.); 3 General Surgery Unit, National Institute of Gastroenterology “Saverio de Bellis”, Research Hospital, Castellana Grotte, 70013 Bari, Italy; fabrizio.aquilino@irccsdebellis.it

**Keywords:** colorectal cancer, laparoscopic surgery, open surgery, obesity, short-term outcomes

## Abstract

**Simple Summary:**

Extended evidence on minimally invasive surgery in colorectal cancer (CRC) settings is needed, especially as applied to obese patients. We aimed to explore and compare postoperative outcomes between open and video-laparoscopic (VL) surgery in two groups of obese patients undergoing surgical resection for CRC. VL surgery was found to reduce postoperative recovery time and the severity of complications. This Italian experience provides a further contribution to the short-term prognostic quality of minimally invasive VL surgery in obese patients.

**Abstract:**

**Background:** Minimally invasive surgery in obese patients is still challenging, so exploring one more item in this research field ranks among the main goals of this research. We aimed to compare short-term postoperative outcomes of open and video-laparoscopic (VL) approaches in CRC obese patients undergoing colorectal resection. **Methods:** We performed a retrospective analysis of a surgical database including 138 patients diagnosed with CRC, undergoing VL (*n* = 87, 63%) and open (*n* = 51, 37%) colorectal surgery. As a first step, propensity score matching was performed to balance the comparison between the two intervention groups (VL and open) in order to avoid selection bias. The matched sample (*N* = 98) was used to run further regression models in order to analyze the observed VL surgery advantages in terms of postoperative outcome, focusing on hospitalization and severity of postoperative complications, according to the Clavien–Dindo classification. **Results:** The study sample was predominantly male (*N* = 86, 62.3%), and VL was more frequent than open surgery (63% versus 37%). The two subgroup results obtained before and after the propensity score matching showed comparable findings for age, gender, BMI, and tumor staging. The specimen length and postoperative time before discharge were longer in open surgery (OS) patients; the number of harvested lymph nodes was higher than in VL patients as well (*p* < 0.01). Linear regression models applied separately on the outcomes of interest showed that VL-treated patients had a shorter hospital stay by almost two days and about one point less Clavien–Dindo severity than OS patients on average, given the same exposure to confounding variables. Tumor staging was not found to have a significant role in influencing the short-term outcomes investigated. **Conclusion:** Comparing open and VL surgery, improved postoperative outcomes were observed for VL surgery in obese patients after surgical resection for CRC. Both postoperative recovery time and Clavien–Dindo severity were better with VL surgery.

## 1. Introduction

Colorectal cancer (CRC) is the third most common malignant neoplasm and the fourth leading cause of death globally, accounting for nearly 1.8 million new cases and 881,000 deaths in 2018 [1]. According to the latest epidemiological projections, by 2030, CRC rates will have risen considerably, especially among younger people [2]. 

Oncologically speaking, obesity [3], reduced physical activity [4,5], Western diet [6], and diabetes [7] are the main factors involved in the increased incidence of CRC. From a surgical perspective, minimally invasive surgery has gradually become a standard treatment for patients diagnosed with colorectal cancer, ever since the first video-laparoscopic (VL) colorectal resection reported by Jacobs in 1991 [8]. 

As obesity has developed into a major social and medical problem worldwide, colorectal surgeons have increasingly been called upon to treat growing numbers of obese patients. There is a recognized association of obesity as a CRC risk factor [9,10], and in this context, for each 5-unit increment in BMI, a recent meta-analysis found a 30% higher risk of CRC in men and 12% higher risk in women [11]. 

Historically, obesity was a contraindication for minimally invasive colorectal surgery [12]. Today, refinements of surgical and anesthetic techniques have been shown to successfully promote VL colorectal surgery even for high-risk subjects, including obesity phenotypes [13]. A recent propensity-matched analysis suggested that VL is a safer and less invasive alternative to open surgery for obese subjects with CRC, demonstrating fewer complications within the first 30 postoperative days [13]. This finding is noteworthy, as increased intra-abdominal visceral fat is a well-known risk factor for technical VL surgery complications, requiring conversion to open surgery [14]. Indeed, obesity makes it difficult to find typical surgical landmarks and to achieve adequate laparoscopic vision. Moreover, an interrelationship has been demonstrated between obesity and increased postoperative adverse events, such as readmission, reintervention, and mortality [12,14,15]. These events are partially due to the higher occurrence of cardiopulmonary comorbidities and wound healing disorders in obese subjects [16,17,18].

However, VL has well-known advantages, including earlier postoperative recovery, reduced pain, early mobilization, lesser surgical trauma, and a better immune status, which may be especially evident in obese patients [17,18]. Nevertheless, there can be no doubt that obesity still poses a surgical challenge, and a scientific consensus about the short-term and oncological outcomes of VL colorectal surgery is still lacking [19]. Among the main factors influencing prognosis patterns, the surgeon’s executive experience plays a critical role, especially regarding total mesocolic or mesorectal excision (Complete Mesocolic Excision (CME) or Total Mesorectal Excision (TME)) and central vascular ligation, as well as adequate lymph node retrieval [20]. Although the literature prescribes VL colorectal surgery as the primary treatment for CRC, even in obese subjects, the results show many forms of bias and confounders that were not controlled for, mainly related to the type of tumor, the age of patients, and staging of the lesions [21].

This research aimed to explore differences in short-term postoperative outcomes between open and VL surgery in a single surgical experience of obese patients diagnosed with CRC.

## 2. Materials and Methods

### 2.1. Study Population and Design

From January 2013 to December 2020, in total, one hundred and thirty-eight consecutive CRC patients were scheduled for laparoscopic or open resection by Dr. Vincenti in two different Surgery Units, i.e., University Hospital “Policlinico” of Bari (Apulia, Southern Italy) and the National Institute of Gastroenterology Research Hospital “Saverio de Bellis” (Castellana Grotte, Apulia, Southern Italy). 

The treatment protocol was based on the National Comprehensive Cancer Network (NCCN) guidelines [22]. Eligibility requirements were a minimum age of 18 years at the time of enrolment, a confirmed diagnosis of colorectal cancer, and a pre-operative condition of obesity (assessed as BMI >/= 30 Kg/m^2^). Exclusion criteria included patients with concurrent emergency conditions (i.e., perforation or occlusion), pregnancy, co-existing peritoneal carcinomatosis, combined operations for other diseases, contraindications for VL surgery (i.e., cardiorespiratory comorbidities), and need for transverse resection or total colectomy. After the lead surgeon’s (L.V.) assessment of the surgical challenge, the allocation to open surgery or VL for each patient depended on the pre-operative anesthesiological risk (i.e., physical evaluation according to the American Society of Anesthesiologists (ASA) criteria), as clinically assessed by an experienced Intensive Care Unit (ICU) specialist. All operations were performed by the same lead surgeon, who had extensive experience in colorectal surgery (L.V.), thus avoiding any risk of operator bias.

The study protocol (ClinicalTrials.gov Identifier: NCT04716062) met the principles in the Declaration of Helsinki and was approved by the Ethics Committee of the National Institute of Gastroenterology “S. De Bellis” Research Hospital (Castellana Grotte, Apulia, Italy). All patients gave written or verbal consent to take part in this study.

### 2.2. Demographic and Clinical Variables

Clinical data collection included both electronic and paper medical records. The information database included age, gender, body weight and height, and body mass index (BMI) data. Anthropometric measurements were taken by a senior nutritionist (R.Z.), with participants dressed in lightweight clothing and without shoes. Variables were all collected at the same time between 07:00 and 10:00 after overnight fasting. Height was measured to the nearest 0.5 cm using a wall-mounted stadiometer (Seca 711; Seca, Hamburg, Germany). Bodyweight was determined to the nearest 0.1 kg using a calibrated balance beam scale (Seca 711; Seca, Hamburg, Germany). BMI was calculated by dividing body weight (Kg) by the square of the height (m^2^), while obesity was classified according to World Health Organization criteria (>= 30.0 kg/m^2^) [23]. The surgical data we gathered consisted of the tumor location (right, left, or rectum), tumor staging (I to IV) and grading (G1 to G3), anastomosis (yes/no), previous abdominal surgery (yes/no), specimen length (cm), operative time (min), harvested lymph nodes (n), circumferential resection margin after proctectomy (Circumferential Resection Margin, CRM) (mm) [24,25], and distal clearance (mm) [26]. Based on the AJCC/TNM classification [27], tumor stages were defined as follows: I, tumor invading submucosa; II, tumor invading muscularis propria; III, tumor invading through the muscularis propria into pericolorectal tissues; IV, tumor penetrating the surface of the visceral peritoneum or tumor directly invading or adherent to surrounding organs or structures. According to histologic criteria, the tumor grading was classified as the highest degree of tumor differentiation (G1, G2, G3) [28]. 

### 2.3. Short-Term Postoperative Outcomes 

As the principal outcome variables of the study, we selected the length of postoperative hospital stay (days) and the severity of postoperative complications, as assessed using the Clavien–Dindo classification [29] and according to the following grading scale: grade I, any deviation from the ordinary postoperative course without the need for pharmacological treatment or surgical, endoscopic, and radiological interventions; grade II, requiring pharmacological treatment including blood transfusions and total parenteral nutrition; grade III, requiring surgical, endoscopic, or radiological intervention; grade IV, life-threatening complication requiring intensive care unit management; grade V, death.

### 2.4. Surgical Procedures 

*Right colectomy.* For the laparoscopic surgery procedure, pneumoperitoneum at 12–14 mmHg was achieved by inserting the Verres needle in the left subcostal position; three trocars were placed. All procedures were conducted with a medial–lateral approach, starting with ligation of the ileocolic vessels at the mesenteric axis origin to ensure adequate lymphadenectomy. Partial omentectomy with central ligation of the gastroepiploic vessels was performed in cases of liver flexure tumors. Bowel reconstruction was performed via intracorporeal mechanical ileo-transverse anastomosis. The specimen was extracted through a Pfannenstiel’s mini-incision, protected by an endobag or steri-drape. 

Open procedures were performed with the same technical rules through a midline laparotomy.

*Left colectomy*. Similarly, the laparoscopic procedure required pneumoperitoneum at 12–14 mmHg to be obtained by inserting the Verres needle in the umbilical position. Three or four trocars were placed, depending on the abdominal conformation, to allow satisfactory exposure of the surgical landmarks. The operation always began with mobilization of the left colic flexure to facilitate a “floppy” anastomosis. The inferior mesenteric vein was identified and ligated to the inferior border of the pancreas. Ligation of the inferior mesenteric artery was performed at the level of the aortic plane, ensuring careful preservation of the hypogastric nerves. An end-to-end transanal colorectal anastomosis was performed according to the Knight–Griffen technique [29]. Open procedures were performed with the same technical rules through a midline laparotomy.

*Rectal resection.* The first steps of laparoscopic rectal resection were comparable to a left colectomy, involving the insertion of four trocars to allow rectal resection. Partial mesorectal excision (PME) was performed for upper rectal tumors, while total mesorectal excision (TME) was considered adequate in the case of middle and lower rectal tumors [30]. The anastomosis was performed according to the Knight–Griffen technique [31] or manually when coloanal anastomosis was required. Ileostomy was always performed for colonanal anastomosis, as well as in cases of total mesorectal excision (TME) in patients with comorbidities or previous neoadjuvant treatment. The Hartmann procedure was performed for locally advanced rectal cancer in patients with multiple comorbidities [32]. The Miles procedure was only undertaken for ultra-low rectal neoplasia with sphincter infiltration [33].

### 2.5. Statistical Analysis

We performed statistical analysis of baseline variables, expressed as means ± standard deviation (SD) for continuous variables and proportions (%) for the frequency of categorical variables. The normality of distribution was assessed for each variable using Shapiro’s test. Spearman’s correlation matrix was built for all continuous pathological and anthropometric variables to check for interrelated variables, in order to avoid collinearity effects in the model. For comparison between VL and open surgery groups, null hypotheses for a number of rejection tests were used, namely the Mann–Whitney sum rank test was used for non-normal distributed continuous variables; an independent samples test was used for normal distributed continuous variables; a chi squared test was used categorical variables; Fisher’s exact test was used for categorical variables with a number lower than 5 observations. *p*-values less than or equal to 0.05 were considered statistically significant, with 95% confidence intervals. 

To balance the group comparisons and avoid selection bias caused by the arbitrary allocation of the patient to a particular type of surgery, a propensity score model was constructed. We used the most common method to estimate the propensity score, a logistic regression model, in which treatment status (VL) is regressed based on observed baseline characteristics. The estimated propensity score is the predicted probability of treatment derived from the fitted regression model. 

Patients in the intervention group (VL) were compared with those in the control group (OS) using nearest neighbor (NN) [34] matching for the main covariates, i.e., age, sex, BMI, and tumor location (right, left, or rectum) and using a caliper of 0.1. In addition to hypothesis tests, the standardized difference or effect size (EF) [35] was used to test the differences between continuous variables in terms of effect size instead of using null hypothesis rejection tests. In line with showing effect size, for comparison between categorical variables (proportions), we used the prevalence differences (open surgery—VL). After matching, we ran a diagnostic balance analysis to assess the performance of matching among the groups. Since the matching balance was not perfect for each variable, we chose to operate regression models in which we also corrected for the same covariates of matching variables [36].

Furthermore, multivariable regression models were run to assess associations with the main short-term outcome variables, namely linear regression models using postoperative hospital stay days as the dependent variables and an ordinal logistic regression model using postoperative complications according to Clavien–Dindo classification as the dependent variables. To assess the association between treatment variables and outcomes regardless of other covariates that could modify the effect, we built three hierarchical nested models, adjusted for different sets of major confounders: (1) raw model using only VL as the covariate; (2) model 1 plus age, sex, and BMI; (3) model 2 plus tumor location; (4) model 3 plus length of specimen, previous surgery, staging, clearance/CRM, harvested lymph nodes, and stoma location. Confounding covariates were chosen according to the classic definition of confounders, by which they must be associated with both exposure (i.e., surgical treatment) and outcome. Due to the massive number of covariates used in the models and to avoid any possible overfitting bias or family-wise errors, we used a Bonferroni correction showing pre and postcorrection *p* values in the tabs. In addition, to compare the fitting of every nested model, we performed the extra sum-of-squares F test for the linear nested models and Pearson chi square multiple comparison for ordinal nested models. 

The methodological approach and analyses were designed and performed by a senior epidemiologist (R.S.) and biostatistician (F.C.) using RStudio software version 1.2.5042.

## 3. Results

### 3.1. Baseline Data

**Figure 1:**
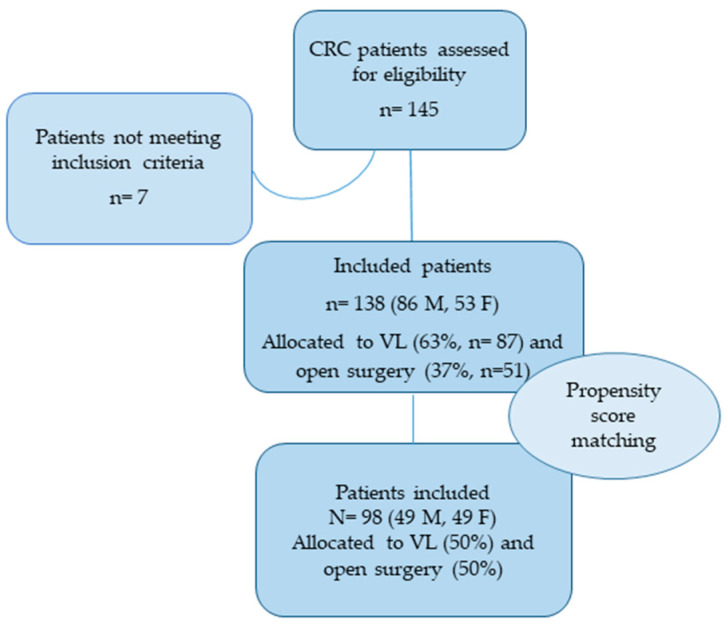
Study flowchart. Abbreviations: Colorectal Cancer (CRC); Video-Laparoscopic Surgery (VL).

The initial sample consisted of 145 patients, 7 of whom were excluded because although initially allocated to VL surgery, due to surgical complications the procedures were converted to open surgery (Figure 1). The remaining sample (*N* = 138) considered for this study featured a majority of males (*N* = 86, 62.3%) with a mean age of 72 ± 9.02 years. The mean BMI was 35.13 ± 5.45 kg/m^2^ for the VL surgery group and 32.98 ± 3.56 kg/m^2^ for the OS group. Fifty-one (37%) subjects underwent OS (48% females, 52% males) and 87 (63%) were assigned to VL surgery (52% females, 48% males). 

### 3.2. Propensity Score Matching

Using the propensity score model, we matched 1:1 to obtain two balanced groups (50% open vs. 50% VL surgery, *N* = 98), as shown in Table 1, panel B. The matching was operated based on major confounding covariates, as previously described in the methods section. Thus, two subgroup analyses were conducted, before (panel A) and after (panel B) the propensity score matching. To assess the balance and then the performance of NN matching, we ran a balance diagnostic analysis (Table 2), showing that rectum tumor locations seemed to be well balanced after the matching. 

Observing effect sizes in Table 1, only the female gender distribution changed after matching, meaning that there were less females in the VL group than the OS group (panel B). 

Tumor staging from G1 to G3 was comparably distributed in the two groups. Subjects with G1-stage CRC were evenly distributed in the two panels, i.e., before and after matching; they were more numerous in OS subjects (22% vs. 20.5% and 20% vs. 29.6% for the OS and VL surgery groups, respectively). Similarly, G3-stage tumors were more prevalent in OS than in VL (*p* < 0.01), even before propensity score matching (34.1% vs. 9% and 35% vs. 8.7% for the OS and LV surgery groups, respectively). Conversely, G2-stage tumors were significantly more common in the VL surgery group (*p* < 0.01), regardless of the application of propensity score matching (43.9% vs. 70.5% and 45% vs. 71.7% for the OS and VL surgery groups, respectively). 

There were no marked differences in the distributions of the tumor location variables between the two groups before matching (EF 0.385, *p* = 1.101); the baseline prevalence of VL surgery in the rectal area was reversed after applying propensity score matching. 

The lengths of the specimens and days of postoperative stay were on average longer in OS patients both before and after matching (EF 0.345 *p* = 0.042 and EF 0.475 *p* = 0.010, respectively), while the number of harvested lymph nodes reached significance against VL only in the matched sample (EF 0.470 *p* = 0.019). In addition, longer times before flatus (EF 0.880 *p* = 0.001 and EF 0.903 *p* = 0.001, respectively, before and after matching), stool canalization (EF 0.660 *p* = 0.001 and EF 0.757 *p* = 0.001, respectively before and after matching), and liquid (EF 0.736 *p* = 0.001 and EF 0.868 *p* = 0.001, respectively before and after matching) and solid (EF 0.804 *p* = 0.001 and EF 1.019 *p* = 0.001, respectively before and after matching) feeding were more likely in OS patients than in the VL group. 

### 3.3. Multivariable Models

Table 3 shows the linear regression analyses performed on hospitalization days as the dependent variables and three different models, all hierarchically nested and stepwise-adjusted for the main confounding covariates. We considered VL surgery as the treatment of interest, since it was negatively associated with the length of hospitalization. Our findings clearly showed that VL-treated patients were more likely to have a 1.89-day shorter hospital stay than OS patients, regardless of major confounding determinants, as specified in footnotes for each model (Table 3).

Similarly, we built an ordinal logistic regression model using postoperative complications according to the Clavien–Dindo classification as the dependent variable, as shown in Table 4. Subjects treated with VL surgery were 67% more likely to have 1 point less of Clavien–Dindo severity than the OS group (OR 0.37, 95%CI 0.14 to 0.95), even after adjustment for major confounders. Furthermore, both regression models showed no significant role for tumor staging in influencing short-term outcomes (95%CI −0.39 to 1.08, *p* 0.36 and 95%CI 0.85 to 2.2, *p* 0.20 for hospitalization days and Clavien–Dindo classification, respectively). Table 5 and Table 6 show multiple testing comparisons between the different nested models, showing good fits for all of them (*p* < 0.05). 

In addition, Table 3 and Table 4 show the additional *p* values after Bonferroni corrections for each covariate used in the different nested models. It is important to highlight that the negative associations between VL surgery and both days of hospitalization and Clavien–Dindo score were weakened in the fully adjusted model (below the statistical significance threshold) by this correction. This was probably due to the large number of covariates (n. 10) and the consequent increase in familywise error. 

It is important to point out that we observed no other analytical model providing evidence of any VL surgery effect on additional postoperative outcomes, including the distal margin (clearance), readmission, and re-intervention, but we chose to not show data to avoid further confusing statements. 

## 4. Discussion

This study was focused on differences in terms of short-term postoperative outcomes in a group of obese subjects affected by CRC, subjected to either VL or open surgery. The major finding was that subjects who underwent VL had a 63% probability of a lower Clavien–Dindo postoperative severity score compared to open surgery subjects, even after adjustment for all confounders. Furthermore, in the models, which had the same confounder control, VL patients experienced a significantly shorter hospitalization time on average than those who underwent open surgery. 

Although it is one of the most common malignant tumors, the pathogenesis and prognosis of CRC are both relatively complex. The present study provides evidence of the reliability of VL surgery as a critical contributor to a better short-term postoperative prognosis for CRC obese patients undergoing surgical resection. Comparative analysis between the OS and VL surgery groups showed a shorter postoperative hospital stay by almost two days and a lower degree of postoperative complications, i.e., one point lower Clavien–Dindo severity, for the group undergoing VL surgery.

Generally speaking, traditional open surgery has long been widely used, due to the many advantages that have accrued over time, including allowing a clear operating area, short operative times, effective removal of tumors and lymph nodes, and a lower recurrence rate. However, the last few decades have seen laparoscopic surgery develop as a viable alternative to the traditional surgical approach. Less pain, faster pulmonary rehabilitation, shorter hospitalization times, better quality of life, and fewer complications related to surgery are among the primary short-term advantages experienced by VL-treated CRC patients [37]. Despite these findings, obesity poses such technical and oncological issues that VL surgery adequacy data may lack consistency. Due to the technical limitations of laparoscopy (i.e., instrumental lack of flexibility), as well as the VL technical experience of surgeons, obese CRC patients have for many years been excluded from the VL approach [10]. In this context, despite some records supporting the practice of VL as equally practicable in obese people, indeed showing some advantages over normal-weight patients [38], an operative consensus has not yet been reached [39]. 

Obesity has been considered a relative contraindication for VL surgery for many years. Besides being a risk factor for cancer itself, excess weight is a major contributor to postoperative morbidity after abdominal surgery. A recent meta-analysis on 5-year oncological outcomes of VL colorectal carcinoma resections comparing obese vs. normal-weight patients showed higher conversion rates and a higher risk of postoperative complications than in the normal-weight group [40]. Despite this, it has already been reported that performing VL abdominal surgery in obese patients can be as safe as with non-obese patients [41]. Furthermore, new evidence has demonstrated certain advantages of VL colorectal resection between obese and non-obese patients [10,12,17]. 

A previous study comparing the short-term surgical outcomes of VL vs. open CRC surgery in the general population, including obese patients, found the VL technique to be associated with longer operative times but less intraoperative blood loss in obese patients [42]. Similarly, another research group analyzed the surgical outcomes of 155 obese patients (BMI >30 Kg/m^2^) and found less intraoperative blood loss but a longer surgical duration in the VL compared to the open surgery group [43]. The longer operative time could be due to technical difficulties in central ligation for adequate lymphadenectomy and mesentery dissection [44]. However, the differences between obese and non-obese patients could decrease during the learning curve after acquiring experience, along with the conversion rate and consequently postoperative morbidity [12,44]. 

Therefore, in light of our findings, it is time to re-examine the concept of obesity as a potential risk factor in VL abdominal surgery, also in view of the extensive changes that have occurred since the advent of interventional laparoscopy. Although this surgery is still challenging, technological advances supplying better vision and advanced devices for hemostasis and dissection, in combination with increasing surgical experience in the mini-invasive field, are radically changing this scenario and demonstrating that VL could be not only comparable, but indeed a better option for the treatment of obese patients with CRC [12,14]. 

The present study provides evidence that laparoscopy offers a reliable approach to CRC obese patients undergoing surgical resection, demonstrating a better short-term postoperative prognosis. Comparative analysis between open and VL surgery showed faster recovery with a shorter average hospital stay by almost two days for the VL group. Additionally, our analysis showed a faster recovery of bowel function (time to first flatus and stool canalization), as well as faster initiation of liquid and solid diets, as compared to open surgery. There is still little evidence of improved short-term outcomes for the obese population subset, although these results are clearly detectable in non-obese patients. Nevertheless, several studies in the literature have described a faster recovery of bowel function and shorter length of stay in obese CRC patients operated on with the VL approach [45]. In 2005, Leroy et al. [46] had already demonstrated no negative impact of the VL approach on the postoperative course applied to obese patients undergoing left colectomy. Delaney et al. [17] further investigated the short-term outcomes in CRC obese patients, comparing the VL and open approaches in a retrospective series of 188 patients (94 in each arm of a case-matched analysis). They concluded that surgical site infection (SSI), cardiopulmonary complications, and anastomotic leakage rates were comparable between the two groups. Moreover, they observed a faster recovery time with a shorter hospital stay in the VL group. Indeed, Delaney et al. showed that the benefits of VL surgery in reducing hospital stays are even more pronounced among obese individuals than what is reported for VL colectomy in general [47]. In line with this, in our experience, the VL group showed a prevalence of subjects with uneventful postoperative course, as well as a lower rate of minor complications (Clavien–Dindo grades I and II). In contrast, the rates of major complications (grades III and V) were comparable between the VL and open surgery groups. Furthermore, no significant differences emerged between the two groups in terms of anastomotic leakage. In fact, despite an increased risk of anastomotic leakage in obese patients reported elsewhere, likely due to their comorbid conditions, the leakage rate does not seem to depend on the surgical approach [12,18,47,48]. Nonetheless, in our experience a lower leakage rate was observed in the VL group, which was also true after matching, although it was not possible to further explore this result, due to the limited number of complications observed.

As far as SSI is concerned, it is well-recognized that excess weight may be a major surgical risk factor, due both to altered immune function with reduced lymphocyte responsiveness and longer operative times compared to normal-weight subjects [49]. In this respect, mini-invasive surgery is associated with a better postoperative immune status and shorter incision length [10,18]. However, we found no differences between the groups in terms of postoperative wound infections, as represented by the Clavien–Dindo assessment scores. This may have been due to the early discharge of the patients undergoing colorectal surgery, since it is estimated that a considerable proportion of SSI occurs postdischarge [18]. 

After matching our dataset, fewer harvested lymph nodes were found for the VL group. The number of harvested lymph nodes is known to be affected by a cluster of factors, i.e., age, cancer site, neoadjuvant therapies, disease stage, type of surgery, the expertise of surgeon and pathologist, pathological features, and surgical resection length [50]. Additionally, it has been argued that the number of lymph nodes could decrease with increasing ASA score and BMI [51]. Nonetheless, the mean number of lymph nodes harvested during CRC surgery in the studied population was more than 12, which is the recommendation reported in the international guidelines [52]. Importantly, the mean distal and circumferential margins in the laparoscopic group were comparable to the open surgery group. These results support the oncological adequacy of the VL approach. Only one RCT has so far indicated that the number of lymph nodes harvested with laparoscopic surgery was lower than with open surgery, however the study sample size was small [53]. Hence, the limited number of patients in the study population has probably affected the reliability of the histopathological results.

Some important study limitations need to be considered. First, there may have been selection bias due to the fact that patients were allocated to the VL or open surgery group based on the subjective clinical judgment of the ICU specialist and the chief surgeon. This judgment depended essentially on each patient’s general health status and may have had a critical confounding effect that we could not have controlled. Secondly, the heterogeneity of patients in terms of baseline clinical features, types and locations of tumor, and age could affect the results, with a massive residual confounding effect that cannot be controlled without precise case selection. Third, the small sample size was definitely a weakness of this study. This limitation showed its Achilles heel in the use of a massive set of covariates (n.10) in the models for such a small sample. This resulted in a high familywise error and an increased overfitting bias for the models (shown after Bonferroni correction).

Furthermore, the efficacy and adequacy of VL versus open surgical approach in excess-weight CRC subjects cannot be strengthened by our data due to the lack of a longitudinal survival assessment. Future long-term data collection may further enhance the existing evidence, so far looking to support laparoscopy as a viable offering for obese CRC patients [54]. However, this is the first study to adopt a dual control approach for confounders. In fact, propensity scores were used to reduce selection bias and a complete set of covariates was used to assess the observed VL association with better postoperative outcomes. Naturally, a well-designed randomized controlled trial could resolve most of the issues due to patient allocation to open or VL surgery. Nonetheless, critical ethical problems prevent the implementation of an intervention study, especially since it is now becoming clear that laparoscopic surgery has better results in terms of patient health and safety. 

## 5. Conclusions

The results of our study demonstrate that VL colectomy shows short-term prognostic safety for the treatment of obese patients with CRC, also after controlling for the major determinants commonly associated with worse outcomes. Obese patients are known to pose a surgical challenge, so this approach should be reserved for experienced surgeons in order to reduce conversion rates and enhance the benefits of mini-invasive procedures in this high risk population. We conclude that the laparoscopic approach shows promise in advanced colorectal surgery in clinical practice. 

## Figures and Tables

**Table 1:** Baseline characteristics for patients before and after propensity score matching. Data are shown as mean ± SD continuous variables and as (%) for proportions.

	Before Matching				After Matching			
	Open	Video	Effect Size	*p* *	Open	Video	Effect Size	*p* *
		Laparoscopic				Laparoscopic		
	Surgery	Surgery			Surgery	Surgery		
Frequency (%)	51 (37.00)	87 (63.00)			49 (50.00)	49 (50.00)		
Age (years)	72 ± 9.02	66.83 ± 10.27	0.535	**0.001**	71.78 ± 9.1	63.12 ± 11.02	0.856	**0.001 ‡**
Sex								
Female	25 (49.00)	27 (31.00)	0.373	**0.029**	24 (49.00)	12 (24.50)	0.525	**0.001**
Male	26 (51.00)	60 (69.00)			25 (51.00)	37 (75.50)		
BMI (Kg/m^2^)	35.13 ± 5.45	32.98 ± 3.56	0.467	**0.001**	35.23 ± 5.52	32.05 ± 2.55	0.741	**0.001**
Grading								
G1	9 (22.00)	16 (20.50)	0.684	**0.001**	8 (20.00)	9 (19.60)	0.702	**0.001**
G2	18 (43.90)	55 (70.50)			18 (45.00)	33 (71.70)		
G3	14 (34.10)	7 (9.00)			14 (35.00)	4 (8.70)		
Tumor location								
Right	19 (37.30)	22 (25.30)		0.101	19 (38.80)	3 (6.10)	1.036	**0.001**
Left	10 (19.60)	31 (35.60)	0.385		9 (18.40)	27 (55.10)		
Rectum	22 (22.43)	34 (39.10)			21 (42.90)	19 (38.80)		
Type of surgery Anastomosis								
Hartmann Technique	4 (7.80)	1 (1.10)	0.398	0.064 a	4 (8.20)	1 (2.00)	0.354	0.219 a
Mile Techniques	1 (2.00)	1 (1.10)			1 (2.00)	--		
Postoperative hospital stay (days)	10.16 ± 7.65	7.07 ± 3.05	0.103	**0.001**	10.16 ± 7.65	6.33 ± 1.25	0.700	**0.001**
Harvested Lymph Nodes (*n*)	19.09 ± 9.4	16.82 ± 9.75	0.237	0.101	19.18 ± 9.49	15 ± 8.23	0.470	**0.019**
Staging								
0	1 (2.50)	--	0.201	0.192 a	1 (2.50)	--	0.538	0.652 a
I	9 (22.50)	24 (31.20)			9 (22–50)	13 (27.70)		
II	12 (30.00)	28 (36.40)			12 (30.00)	17 (36.20)		
III	14 (35.00)	23 (29.90)			14 (35.00)	15 (31.90)		
IV	4 (10.00)	2 (2.60)			4 (10.00)	2 (4.30)		
V	--	--			--	--		
Operative time (min)	140.11 ± 50.80	144.94 ± 42.33	0.103	0.332	139.22 ± 51.01	147.81 ± 41.84	0.184	0.192
Previous surgery (yes)	30 (58.80)	50 (57.50)	0.027	0.871	28 (57.10)	24 (49.00)	0.164	0.411
Readmission (yes)	6 (11.80)	10 (11.50)	0.008	0.962	6 (12.20)	5 (10.20)	0.065	0.742
Re-intervention (yes)	7 (13.70)	19 (21.80)	0.213	0.231	6 (12.20)	13 (26.50)	0.367	0.073
Length of Specimen (cm)	34.11 ± 13.8	29.85 ± 10.69	0.345	**0.042**	34.18 ± 13.95	28.84 ± 7.65	0.475	**0.010**
Clearance (cm)	6.62 ± 6.60	5.35 ± 4.02	0.232	0.991	6.71 ± 6.66	5.11 ± 4.12	0.290	0.742
CRM (<1 mm)	2 (10.50)	2 (6.70)	0.138	0.623	2 (11.10)	2 (11.10)	0.001	0.901
Time to flatus (days)	3.12 ± 1.08	2.31 ± 0.74	0.880	**0.001**	3.12 ± 1.08	2.3 ± 0.68	0.903	**0.001**
Time to canalization								
Anastomosis (days)	4.59 ± 1.31	3.77 ± 1.16	0.660	**0.001**	4.59 ± 1.31	3.67 ± 1.12	0.757	**0.001**
Stoma (days)	2.27 ± 1.27	1.57 ± 0.728	0.683	0.082	2.27 ± 1.27	1.47 ± 0.8	0.755	**0.039**
Liquid oral diet (days)	2.77 ± 1.2	1.96 ± 0.99	0.736	**0.001**	2.77 ± 1.2	1.86 ± 0.89	0.868	**0.001**
Solid oral diet (days)	4.41 ± 1.24	3.45 ± 1.15	0.804	**0.001**	4.41 ± 1.24	3.2 ± 1.12	1.019	**0.001**
Postoperative complications								
Dehiscence (yes)	11 (21.60)	5 (5.70)	0.473	**0.001**	10 (20.40)	2 (4.10)	0.514	**0.001**
(Clavien–Dindo)	1.36 ± 1.37	0.77 ± 1.37	0.471	**0.010**	1.22 ± 1.22	0.63 ± 0.95	0.538	**0.021**
0	19 (40.40)	51 (60.00)	0.505	0.152 a	19 (42.20)	32 (65.30)	0.556	0.14 a
I	5 (10.60)	10 (11.80)			5 (11.10)	5 (10.20)		
II	15 (31.90)	19 (22.40)			15 (33.30)	10 (20.40)		
III	4 (8.50)	2 (2.40)			4 (8.90)	2 (4.10)		
IV	3 (6.40)	3 (3.50)			2 (4.40)	--		
V	1 (2.10)	--			--	--		

Effect Size: Standardized differences for continuous variables and prevalence differences for categorical variables; * Mann–Whitney sum rank test used where not otherwise specified; ‡ independent samples *t* test. Chi squared test used for categorical variables where not otherwise specified; a Fisher’s exact test. Significance shown in bold.

**Table 2:** Balance measures of propensity score matching.

Matching Variables	Type	Diff. Adj.	M. Threshold
Age (years)	Continue	−0.8422	Not Balanced, >0.1
Sex (Male)	Binary	0.2449	Not Balanced, >0.1
BMI (Kg/m^2^)	Continue	−0.8955	Not Balanced, >0.1
Tumor Location			
Right	Binary	−0.3265	Not Balanced, >0.1
Rectum	Binary	−0.0408	Balanced, <0.1
Left	Binary	0.3673	Not Balanced, >0.1

Legend: Type refers to the types of variables. Diff. Adj.: the (standardized) difference in means between the two groups after matching; M. Threshold: threshold for mean differences.

**Table 3:** Linear regression model for hospitalization (days).

Covariates	Coefficient	Std. Err.	CI 95%	*p*	*p*-Adjusted “Bonferroni”
Model 1					
Treatment (VL)	−3.09	0.93	−4.91 to −1.28	**0.001**	**0.011**
Model 2					
Treatment (VL)	−2.84	1.00	−4.81 to −0.88	**0.005**	**0.019**
Age (years)	0.03	0.05	−0.06 to 0.12	0.525	0.992
Sex (Male)	−0.84	0.98	−2.76 to 1.09	0.396	0.993
BMI (Kg/m^2^)	−0.02	0.11	−0.23 to 0.19	0.855	0.992
Model 3					
Treatment (VL)	−3.09	0.99	−5.02 to −1.15	**0.002**	**0.012**
Age (years)	0.05	0.05	−0.04 to 0.13	0.323	0.993
Sex (Male)	−0.87	0.96	−2.75 to 1.02	0.369	0.992
BMI (Kg/m^2^)	−0.01	0.11	−0.21 to 0.20	0.958	0.991
Location (Rectum)	2.91	1.06	0.83 to 4.99	**0.006**	**0.039**
Location (Left)	2.72	1.17	0.43 to 5.02	**0.021**	0.141
Model 4					
Treatment (VL)	−1.89	0.79	−3.44 to −0.33	**0.019**	0.241
Age (years)	0.03	0.03	−0.03 to 0.10	0.348	0.992
Sex (Male)	−1.06	0.73	−2.48 to 0.36	0.147	0.991
BMI (Kg/m^2^)	−0.08	0.08	−0.24 to 0.07	0.286	0.072
Location (Rectum)	2.70	0.97	0.80 to 4.59	**0.006**	0.991
Location (Left)	1.45	0.91	−0.33 to 3.23	0.113	0.992
Length of Specimen (cm)	−0.01	0.03	−0.07 to 0.05	0.709	0.992
Previous surgery (yes)	0.29	0.66	−1.00 to 1.58	0.662	0.993
Staging	0.35	0.37	−0.39 to 1.08	0.369	0.992
Clearance < 1 cm/CRM < 1 mm	−1.67	1.27	−4.17 to 0.82	0.191	0.991
Harvested Lymph nodes (n)	0.03	0.04	−0.05 to 0.11	0.444	0.993
Ileostomy	0.59	0.95	−1.28 to 2.46	0.535	0.994
Colostomy	−1.03	1.86	−4.67 to 2.62	0.582	0.992

Model 1: raw model; model 2: corrected for age, sex, BMI; model 3: corrected for age, sex, BMI, and tumor location; model 4: corrected for age, sex, BMI, tumor location, length of specimen, previous surgery, staging, clearance/CRM, harvested lymph nodes, and stoma location. Significance shown in bold.

**Table 4:** Ordinal logistic regression model based on Clavien–Dindo classification.

Covariates	OR	CI 95%	*p* *	*p*-Adjusted “Bonferroni”
	Model 1			
Treatment (VL)	0.41	0.20 to 0.81	**0.011**	**0.012**
	Model 2			
Treatment (VL)	0.41	0.19 to 0.86	**0.019**	0.072
Age (years)	0.99	0.96 to 1.03	0.980	0.992
Sex (Male)	1.13	0.55 to 2.35	0.733	0.991
BMI (Kg/m^2^)	1.01	0.93 to 1.09	0.714	0.993
	Model 3			
Treatment (VL)	0.35	0.16 to 0.76	**0.008**	**0.050**
Age (years)	1.01	0.97 to 1.04	0.777	0.991
Sex (Male)	1.15	0.56 to 2.40	0.702	0.992
BMI (Kg/m^2^)	1.02	0.94 to 1.09	0.690	0.993
Location (Rectum)	2.20	0.97 to 5.10	0.060	0.362
Location (Left)	2.25	0.89 to 5.82	0.086	0.513
	Model 4			
Treatment (VL)	0.37	0.14 to 0.95	**0.041**	0.531
Age (years)	0.99	0.95 to 1.03	0.625	0.992
Sex (Male)	0.67	0.28 to 1.62	0.381	0.993
BMI (Kg/m^2^)	1.01	0.92 to 1.09	0.912	0.992
Location (Rectum)	2.65	0.82 to 8.87	0.106	0.991
Location (Left)	2.18	0.69 to 7.25	0.190	0.993
Length of Specimen (cm)	0.99	0.96 to 1.03	0.856	0.994
Previous surgery (yes)	1.78	0.79 to 4.09	0.162	0.993
Staging	1.35	0.84 to 2.19	0.205	0.992
Clearance < 1 cm/CRM < 1 mm	0.17	0.02 to 0.88	0.062	0.681
Lymph nodes	1.02	0.97 to 1.07	0.326	0.993
Ileostomy	2.14	0.71 to 6.56	0.177	0.994
Colostomy	4.81	0.55 to 41.39	0.147	0.993

Model 1: raw model; model 2: corrected for age, sex, and BMI; model 3: corrected for age, sex, BMI, and tumor location; model 4: corrected for age, sex, BMI, tumor location, length of specimen, previous surgery, staging, clearance/CRM, harvested lymph nodes, and stoma location. Significance shown in bold.

**Table 5:** Model fitting comparison test using a multiple linear regression approach.

Models	Res. Df.	RSS	Df.	Sum of Sq.	F	Pr (>F)
Restricted model	135	3912.4				
Model 1	134	3612.3	1	300.12	11.133	**0.001**
Restricted model	135	3912.4				
Model 2	131	3580.1	4	332.29	3.039	**0.019**
Restricted model	135	3912.4				
Model 3	129	3357.2	6	555.19	3.555	**0.002**
Restricted model	111	1335.1				
Model 4	98	1068.9	13	266.18	1.877	**0.041**

Model 1: raw model; model 2: corrected for age, sex, and BMI; model 3: corrected for age, sex, BMI, and tumor location; model 4: corrected for age, sex, BMI, tumor location, length of specimen, previous surgery, staging, clearance/CRM, harvested lymph nodes, and stoma location. Legend: Res. Df: residual degrees of freedom; RSS: residual sum of squares; Df.: degrees of freedom; F: F test value; Pr(>F): *p* value of F distribution. Significance shown in bold.

**Table 6:** Model fitting comparison test using multiple logistic ordinal regression approach.

Models	Res. Df.	Df.	Chisq.	Pr (Chisq)
Restricted model	127			
Model 1	126	1	6.413	**0.001**
Restricted model	127			
Model 2	123	4	6.594	0.15
Restricted model	127			
Model 3	121	6	10.376	0.10
Restricted model	108			
Model 4	95	13	17.214	0.18

Model 1: raw model; model 2: corrected for age, sex, and BMI; model 3: corrected for age, sex, BMI, and tumor location; model 4: corrected for age, sex, BMI, tumor location, length of specimen, previous surgery, staging, clearance/CRM, harvested lymph nodes, and stoma location. Legend: Res.Df.: residual degrees of freedom; Df.: degrees of freedom; Chi: chi squared test value; Pr (Chisq): *p* value of chi squared distribution. Significance shown in bold.

## Data Availability

The data presented in this study are available on request to the corresponding author.
